# Supplementary material for: Desynchronized liquid crystalline network actuators with deformation reversal capability
Source: Nat Commun. 2021 Jan 27;12:624. doi: 10.1038/s41467-021-20938-6 (PMC7840968; doi:10.1038/s41467-021-20938-6)
Supplement: Supplementary file 1 — Supplementary information [file 41467_2021_20938_MOESM1_ESM.pdf]

Supplementary Information

Desynchronized Liquid Crystalline Network Actuators with Deformation Reversal Capability

Yao-Yu Xiao[1], Zhi-Chao Jiang[1], Jun-Bo Hou[1] & Yue Zhao[1*]

[1]Departement de chimie, Universite de Sherbrooke, Sherbrooke, Quebec, J1K 2R1, Canada.

*Correspondence and requests for materials should be addressed to Y.Z. (email: yue.zhao@usherbrooke.ca)

Supplementary Figures

1. Effect of chain crosslinking on LC-isotropic phase transition of unstretched liquid crystalline polymer

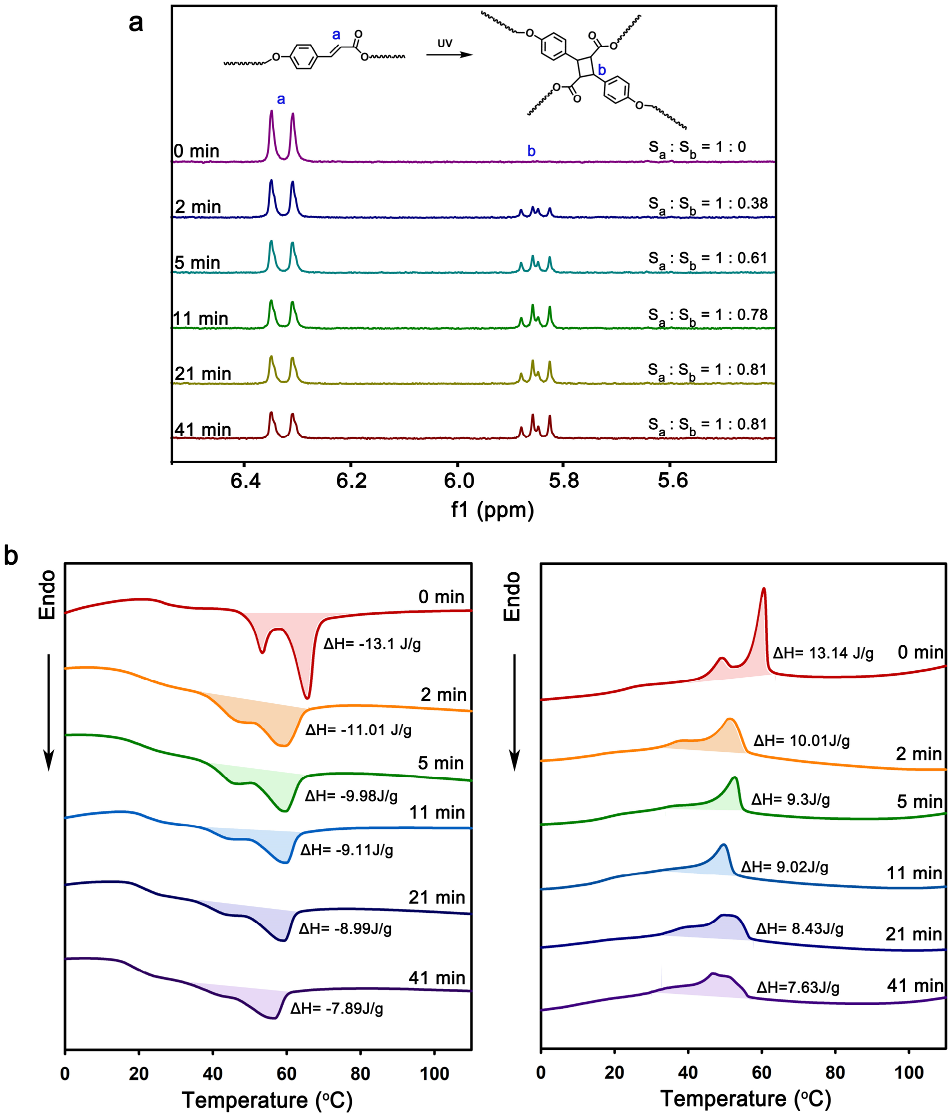

Supplementary Figure 1 |. (a) ^{1}H NMR spectra of liquid crystalline polymer/$CDCl_3$ (concentration: 10 mg ml^{-1}) with different UV irradiation times for polymer chain crosslinking. The increase in the integral of peaks b (S_b) with respect to the integral of peaks a (S_a) indicates increased photodimerization and thus chain crosslinking. **(b)** Differential scanning calorimetry (DSC) heating and cooling curves for LCNs crosslinked in chloroform as in (a). The UV light intensity used in this experiment is 3.5 mW cm^{-2}.

2. Deformation reversal behavior of $LCN_1(X,Y)$

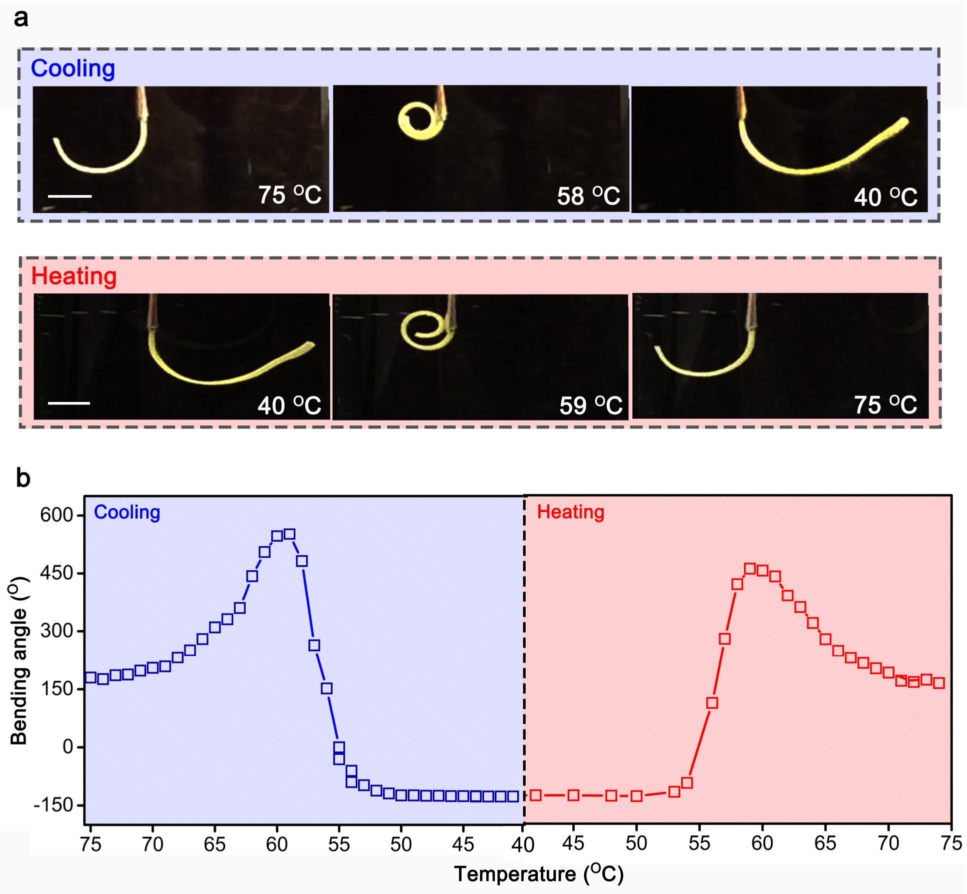

Supplementary Figure 2 |. (a) Deformation reversal behavior of $LCN_1(20,120)$ during a cooling and heating cycle, and **(b)** the corresponding bending angle variations as a function of temperature during cooling and heating processes. The temperature change rate is ca. 2 °C min^{-1}. Scale bars: 2 mm.

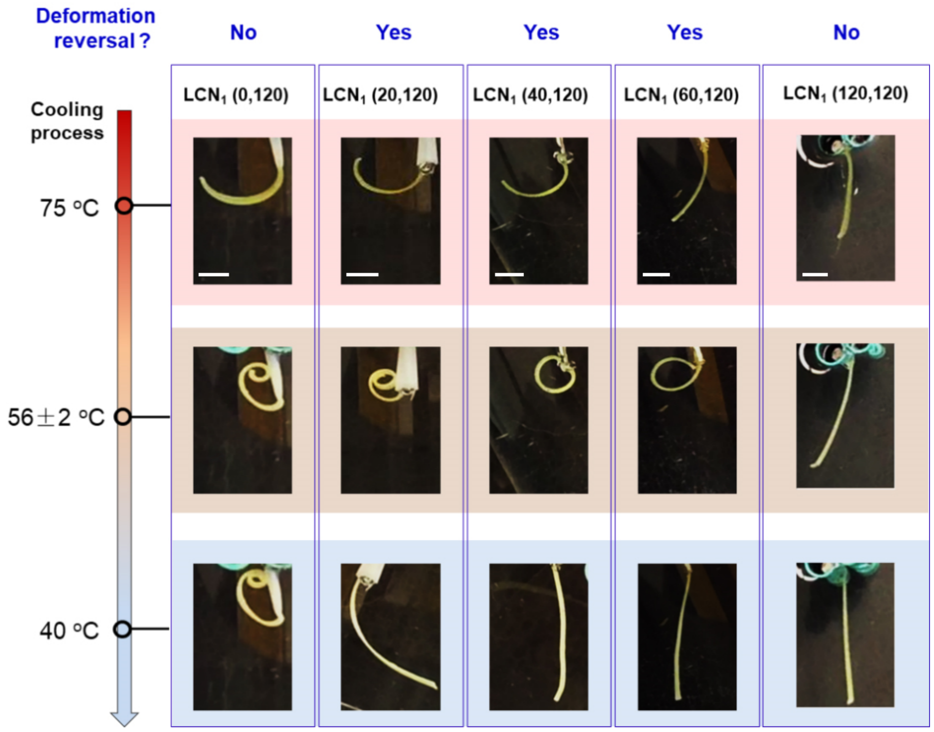

Supplementary Figure 3 |. Photographs showing deformation of LCN_1 with different cross-linking time of side A and the same crosslinking time of side B (120 min) during cooling from 75 °C to 40 °C within ~8 min (scale bars: 2 mm).

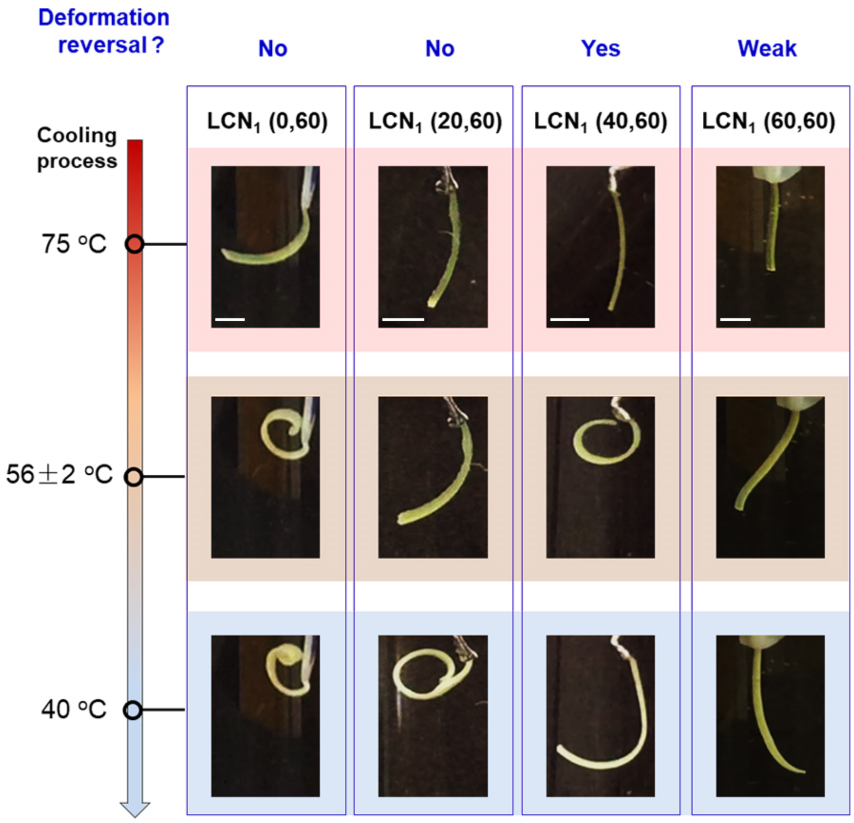

Supplementary Figure 4 |. Photographs showing shape changes of LCN_1 with different crosslinking time of side A while crosslinking time of side B is fixed as 60 min for all samples during cooling (scale bars: 2 mm).

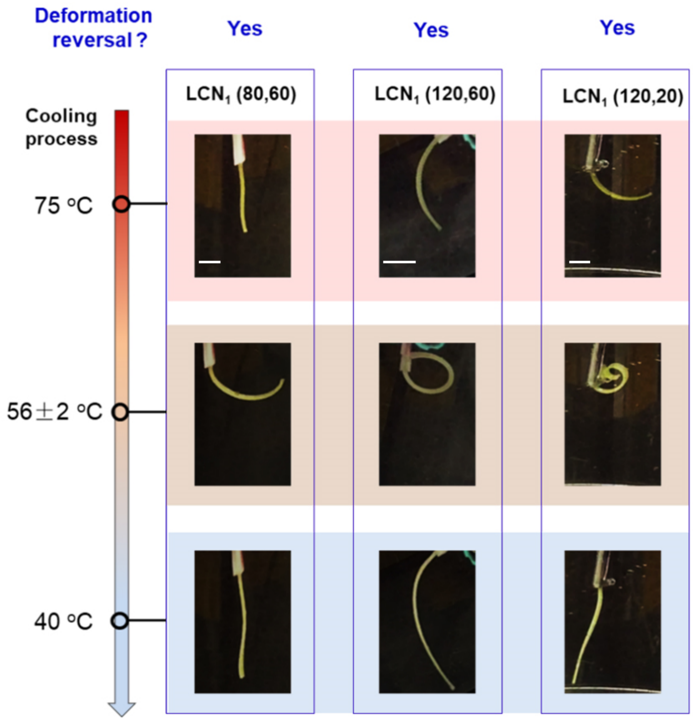

Supplementary Figure 5 |. Photographs showing shape changes of LCN_1(80,60), LCN_1(120,60) and LCN_1(120,20) during cooling. When X (crosslinking time of side A) is smaller than Y (crosslinking time of side B), as shown in Supplementary Figure 3 and 4, the deformation process can be described by $\alpha_1>0$, $d\alpha_I>0$, $d\alpha_{II}<0$. When X is larger than Y, the shape change process is reversed and can be described by $\alpha_1<0$, $d\alpha_I<0$, $d\alpha_{II}>0$, as shown in Supplementary Figure 5 (scale bars: 2 mm).

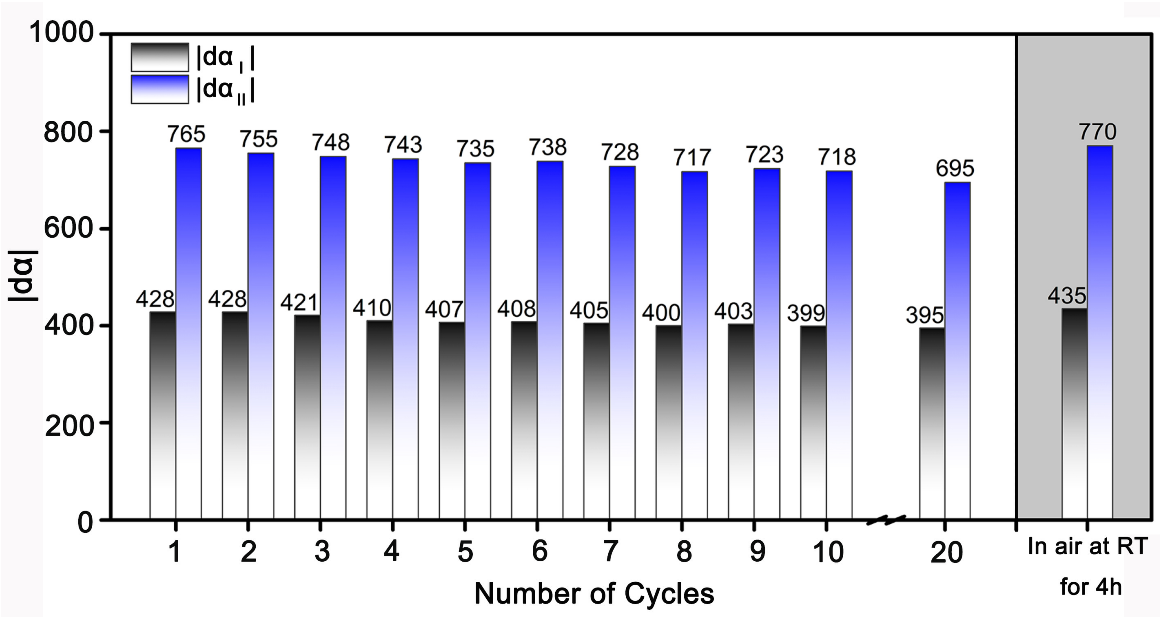

Supplementary Figure 6 |. Change of bending angle during deformation I ($|d\alpha_I|$) and deformation II ($|d\alpha_{II}|$) of LCN_1(20,120) showing the deformation reversal for 20 successive cooling processes (from 75 ºC to 40 ºC in water within ca. 8 min). Only the $|d\alpha_I|$ and $|d\alpha_{II}|$ during cooling processes were recorded to showcase the stable and reversible actuation behavior because: 1) When heating water under static conditions (stirring is not desired, because it will affect the observation of deformation), it is easy to generate obvious temperature gradient and cause water flow due to thermal convection, both of which can interfere with the observation of the deformation process. 2) Reducing the heating rate can basically overcome the above technical problems and realize the observation of the deformation process (as shown in Supplementary Figure 2). However, in this case, each heating process is very long (for example, one heating and cooling cycle shown in Supplementary Figure 2 could take more than 35 min), thus, it is inconvenient to record data for tens of deformation cycles.

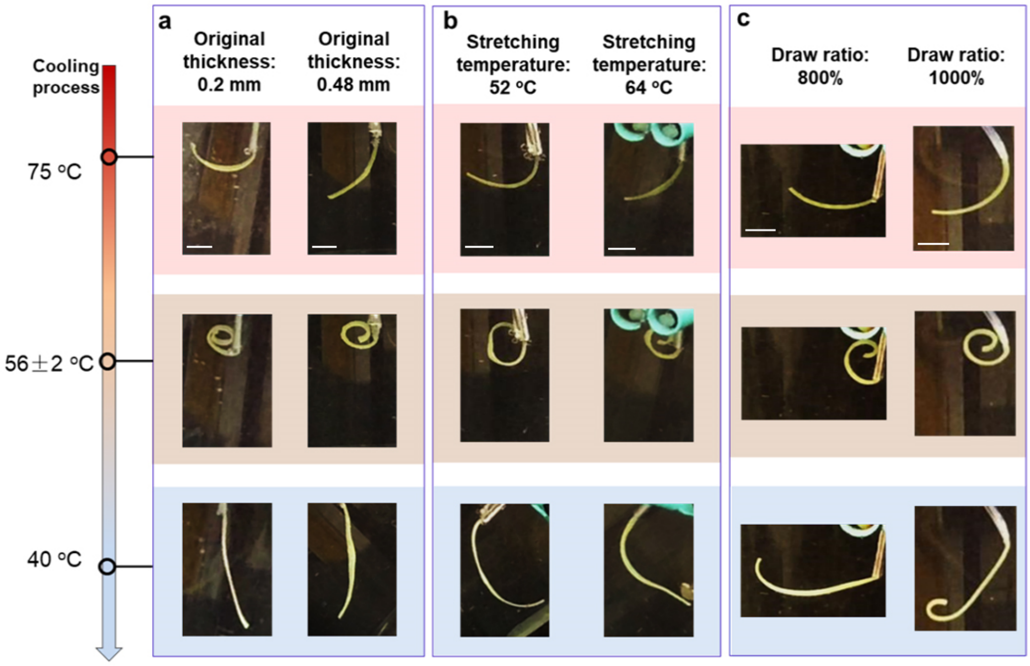

Supplementary Figure 7 |. Photographs showing deformation reversal behaviors of LCN1(20,120) with different **(a)** original film thickness and **(b)** stretching temperature, recorded upon cooling. **(c)** Deformation reversal behaviors of LCN actuators stretched to 800% and 1000% before having their side A and side B crosslinked for 20 min and 120 min, respectively (scale bars: 2 mm).

3. Variable-temperature X-ray diffraction measurements

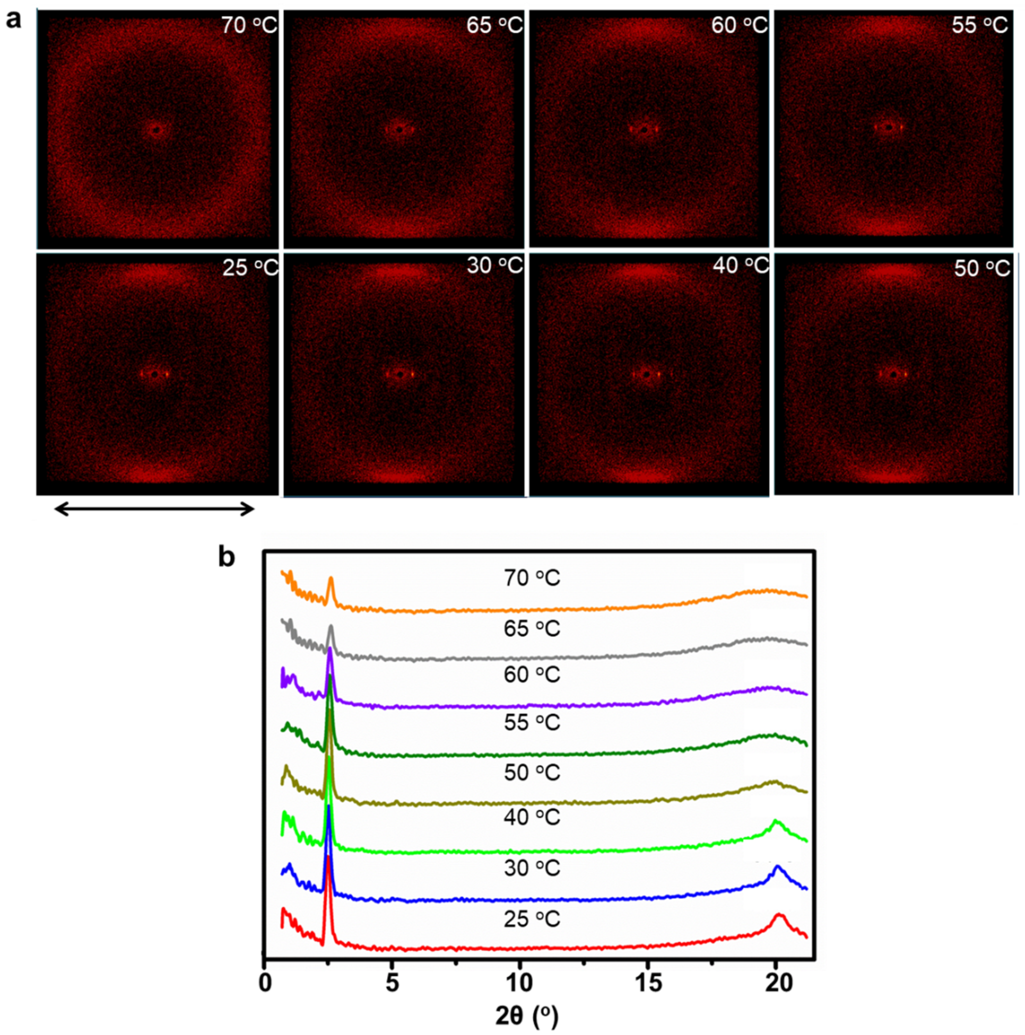

Supplementary Figure 8 |. (a) Two-dimensional XRD patterns and **(b)** one-dimensional XRD patterns of a uniformly crosslinked LCN (draw ratio ~350%, 320 nm UV light, intensity 80 mW cm^{-2}, photocrosslinking in the smectic phase for 1 h per side, thickness 0.07 mm) at different temperatures on cooling from 70 to 25 °C. The black arrow in (a) indicates the stretching direction of the sample.

4. Coumarin-LCN actuator with nematic phase

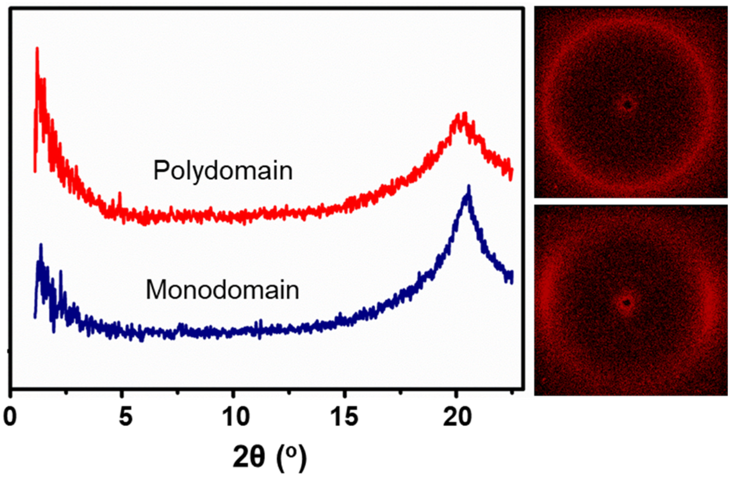

Supplementary Figure 9 |. Two-dimensional XRD patterns and one-dimensional XRD patterns of polydomain coumarin-LCP (liquid crystalline polymer, uncrosslinked) and monodomain coumarin-LCN (crosslinked) at 25 °C.

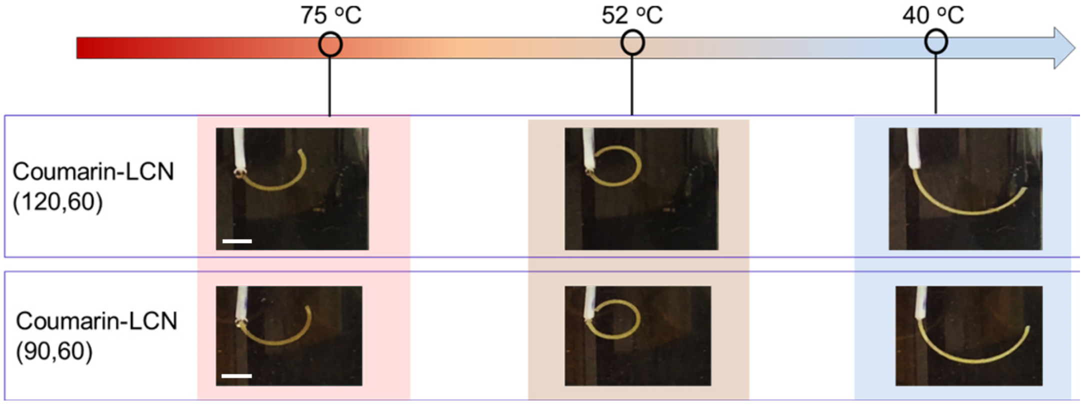

Supplementary Figure 10 |. Photographs showing deformation reversal behavior of coumarin-LCN actuators with only a nematic phase during cooling process (scale bars: 2 mm).

5. Gel fraction and SEM of LCNs with different photocrosslinking times

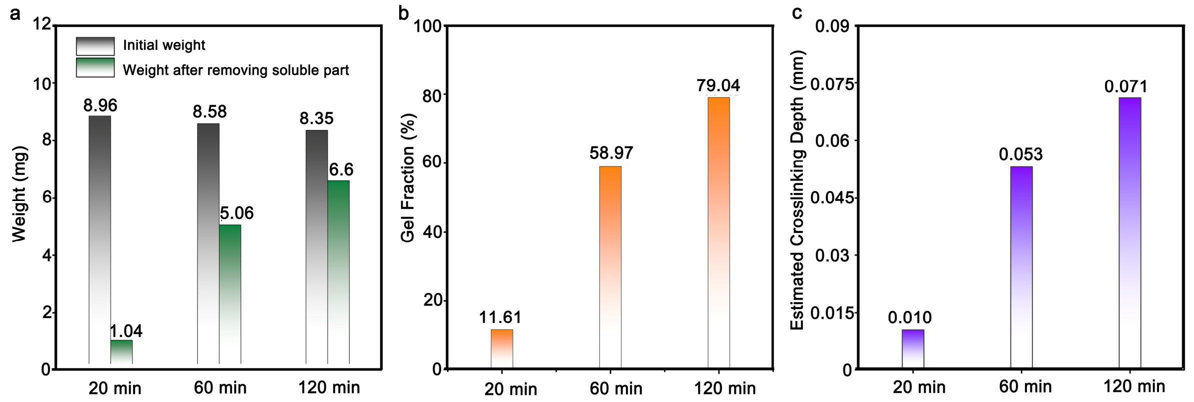

Supplementary Figure 11 |. (a) Weights of the samples with different crosslinking times measured before and after removing the uncrosslinked part, denoted as w_0 and w_1, respectively. The original stretched film with a thickness (*d*) of 0.09 mm was crosslinked for only one side for 20 min, 60 min, and 120 min respectively. Then, the samples were immersed in 50 ^{o}C chloroform (30 ml) to remove the soluble part; the removal process was repeated three times before drying. **(b)** Gel fraction of LCNs (calculated by w_1/w_0) with different crosslinking times. **(c)** Estimated crosslinking depth in the three crosslinking situations. The crosslinking depth is calculated by multiplying the initial thickness of the stretched sample (0.09 mm) by the gel fraction (this is an approximation by assuming that all crosslinked chains remained in the solution regardless of the crosslinking density, and that the polymer density did not change upon crosslinking). As such, the estimated crosslinked layer are 0.010 mm, 0.053 mm and 0.071 mm in the LCNs with one side crosslinked for 20 min, 60 min and 120 min, respectively.

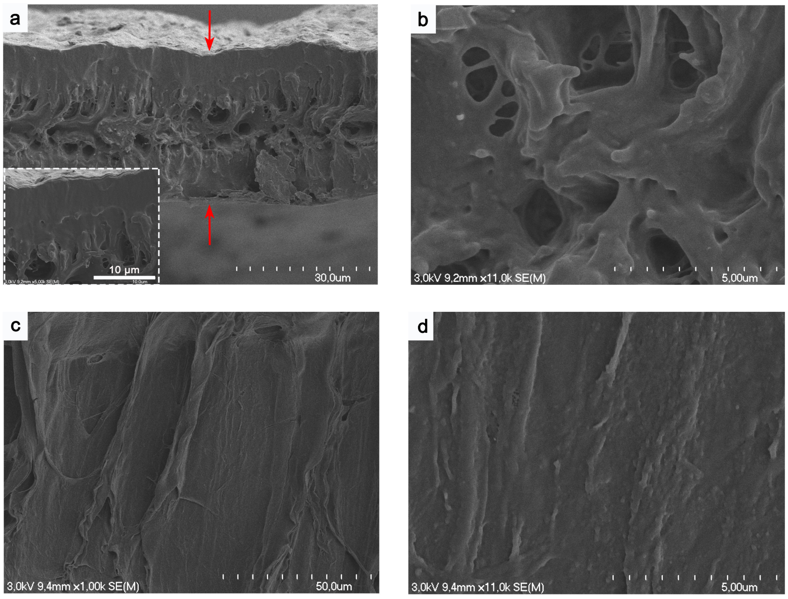

Supplementary Figure 12 | SEM images of the cross-section areas of LCNs with different crosslinking time after removing the soluble parts using the same procedures as mentioned above. (**a, b**). SEM images of the cross-section area of the LCN with both sides photocross-linked for 20 min, showing apparently porous regions in the middle due to the removed un-crosslinked portion. (**c, d**). SEM images of the cross-section area of the LCN with both sides photocrosslinked for 120 min, showing a denser middle region that is likely indicative of a more thorough and uniform crosslinking along the thickness direction. The samples used have a thickness of 0.16 mm during photocrosslinking.

6. Variable-temperature tensile tests and Young's modulus measurements

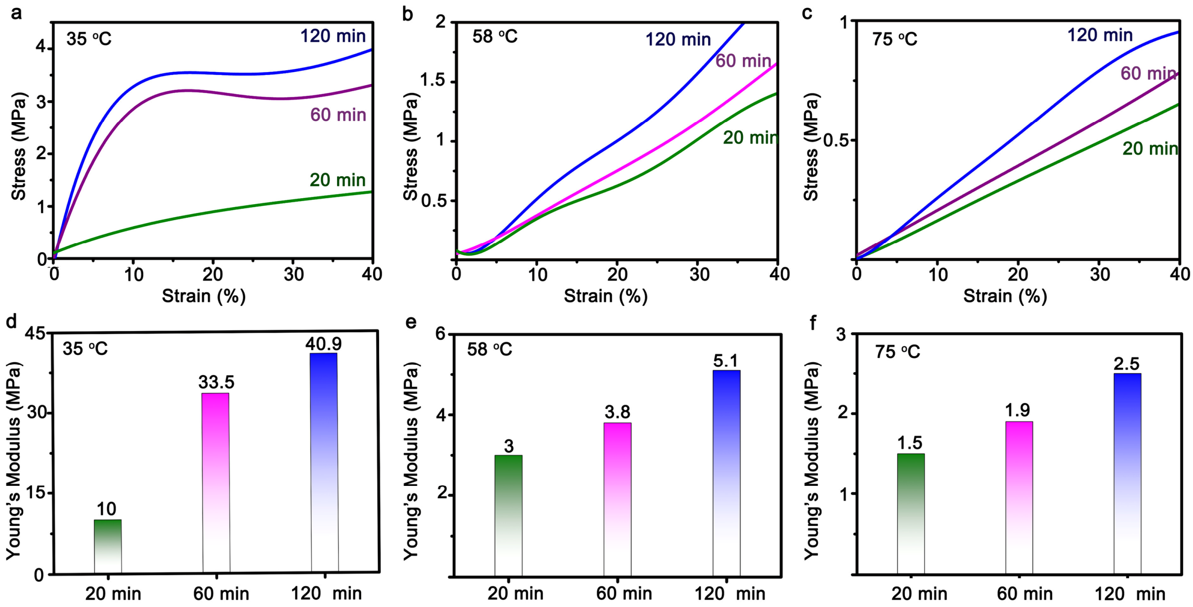

Supplementary Figure 13| (a-c) Tensile test curves of LCN strips with different photocross-linking time for each side, recorded at 35 ºC, 58 ºC and 75 ºC. (**d-f**) Young's modulus of LCN strips with different photocrosslinking time for each side measured at 35 ºC, 58 ºC and 75 ºC.

7. Deformation reversal of LCN2(M,N)

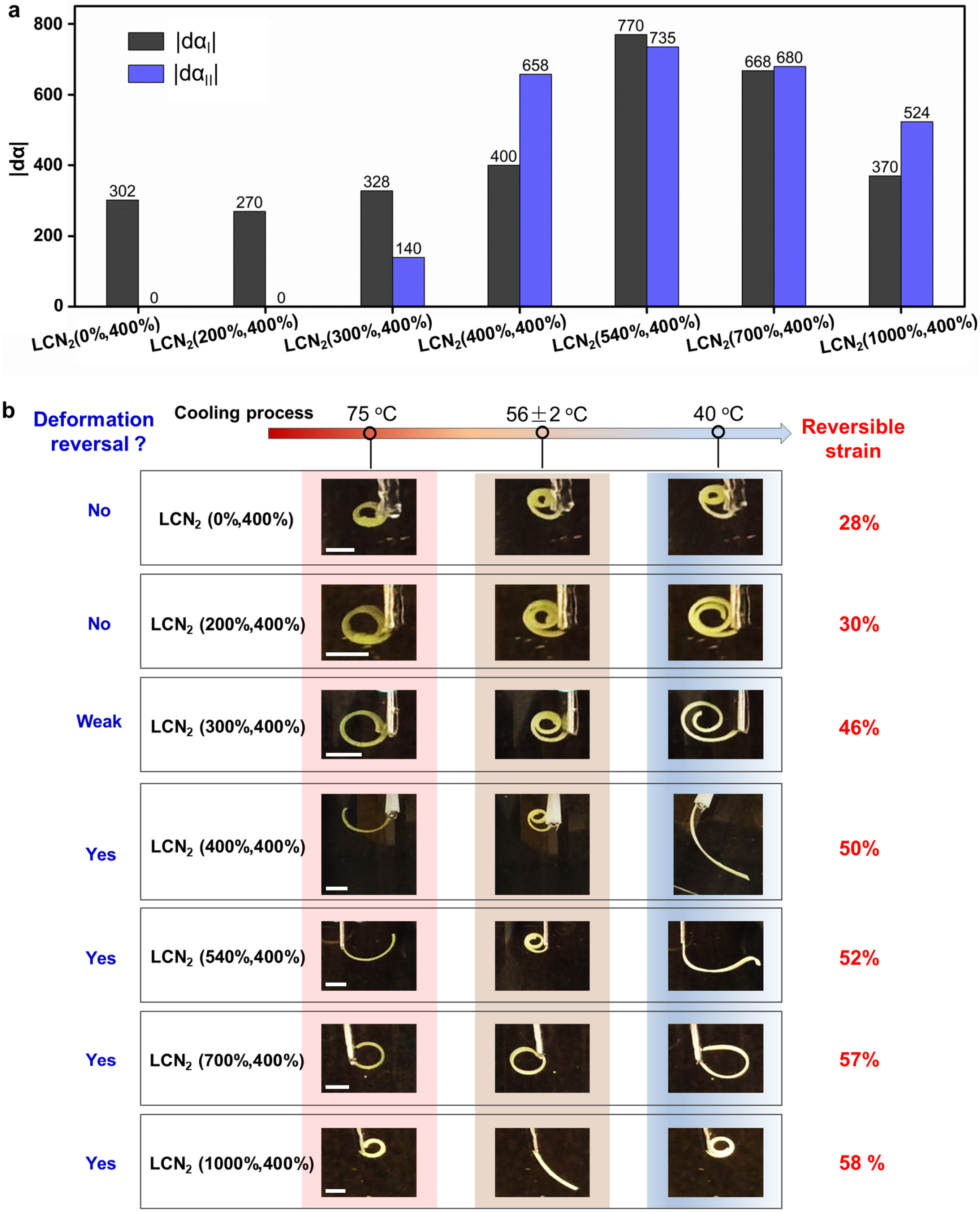

Supplementary Figure 14 |. (a) Change of bending angle (|dα|) during deformation reversal process of LCN2(M,N) upon cooling. **(b)** Photographs showing deformation of LCN2(M,N) during cooling. The side B of all the films possess the same processing condition: λ=400% and crosslinking time of 120 min. For the side A, the crosslinking time is fixed as 20 min, while the λ varies from 0% to 1000% (scale bars: 2 mm).

8. Deformation reversal of in-plane bending

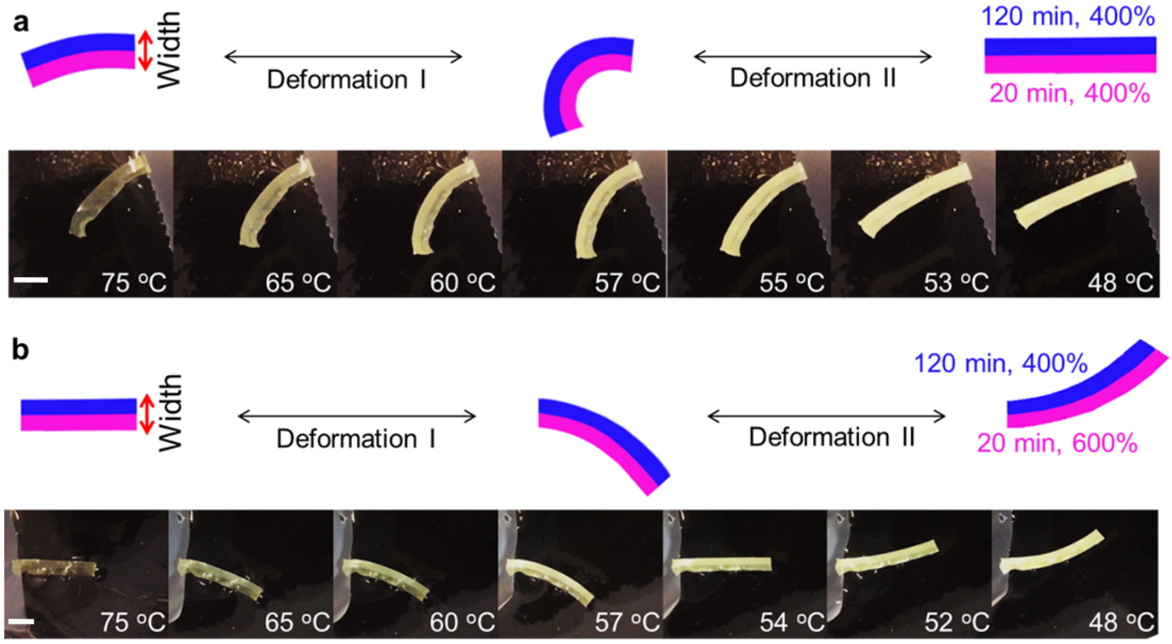

Supplementary Figure 15 |. (a) Schematics and photographs showing the in-plane deformation reversal behavior of the LCN prepared by crosslinking the two halves (along the width of the film) of a uniaxially aligned liquid crystalline polymer (LCP) film for 120 min and 20 min respectively, noting that the two sides of the film along the thickness direction are crosslinked evenly. **(b)** Schematics and photographs illustrating the in-plane deformation of the LCN with asymmetric processing of its two halves along the width direction: one is crosslinked for 120 min at λ=400% and the other is irradiated for 20 min at λ=600% (scale bars: 4 mm).

9. Characterization of polydopamine-coated LCN

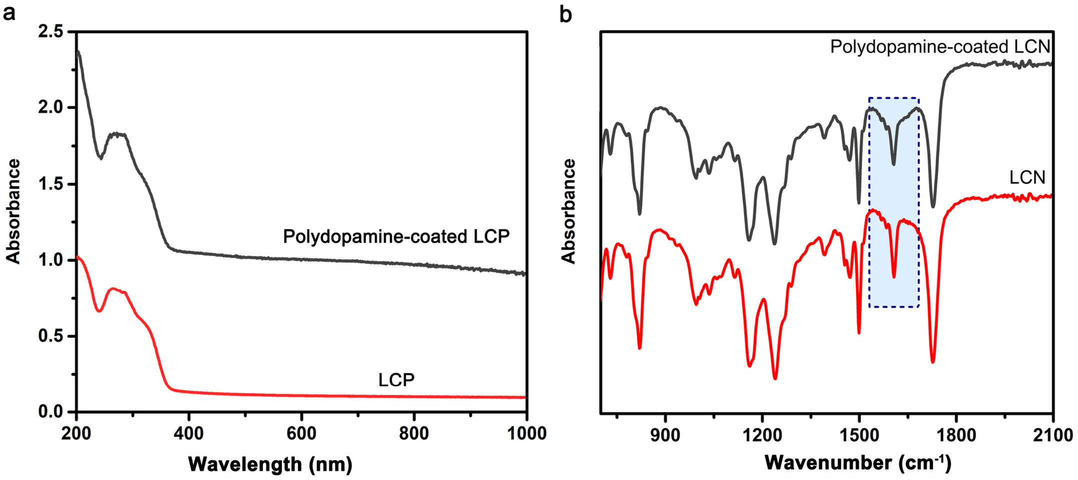

Supplementary Figure 16 |. (a) UV-Vis spectra of thin polymer film of liquid crystalline polymer (LCP) spin-coated on quartz and polydopamine-coated LCP. The spectra show that the polydopamine coating exhibits strong absorption in the visible light and near infrared range. **(b)** ATR-FTIR spectra of LCN and polydopamine-coated LCN. Compared with the pure LCN sample, the absorption peak at about 1600 cm^{-1} of the polydopamine-coated LCN becomes broad due to the increasing amount of C=O groups (ref. S1).

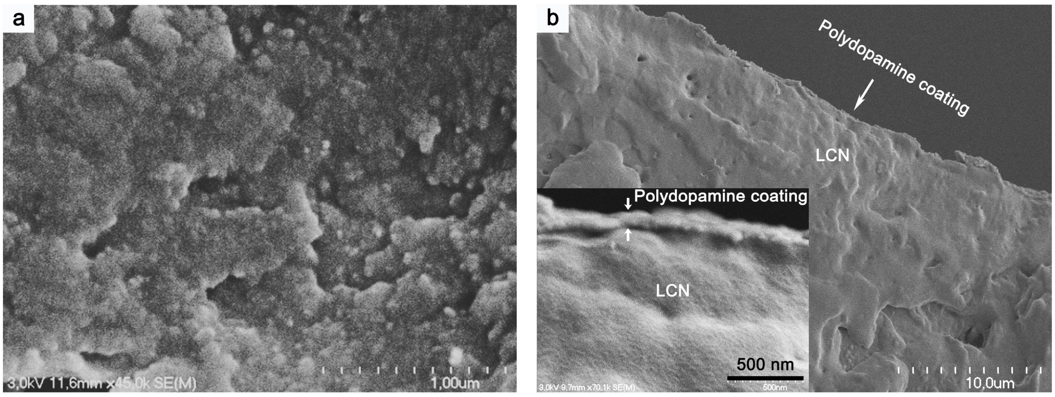

Supplementary Figure 17 |. SEM images for (a) top-view, and (b) cross-section of polydopamine-coated LCN. The average thickness of the polydopamine layer is ca. 130 nm.

10. Deformation reversal of polydopamine-coated LCN during cooling in water

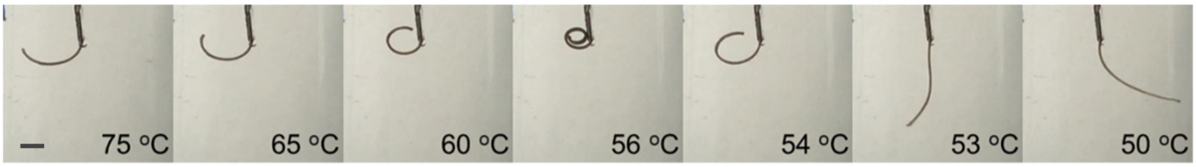

Supplementary Figure 18 |. Photographs showing the deformation reversal behavior of a polydopamine-coated $LCN_1(20,120)$ actuator during cooling in water. The shape evolution is the same as for the $LCN_1(20,120)$ without coating in Figure 2a, indicating that the polydopamine coating has no effect on the actuation behavior of LCN actuators (scale bar: 2 mm).

11. Photothermal effect of the polydopamine-coated LCN actuators

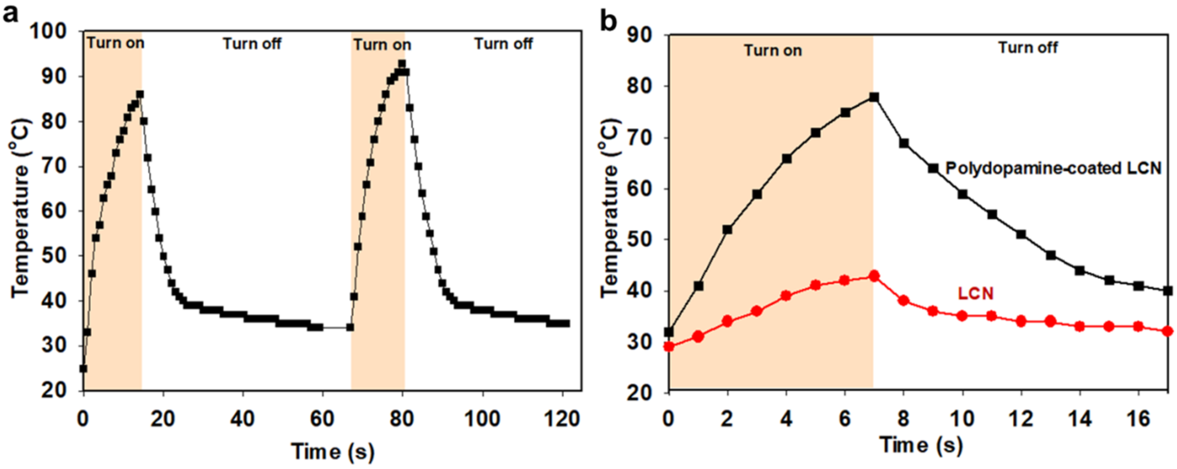

Supplementary Figure 19 |. (a) Temperature variation of polydopamine-coated LCNs during on/off NIR light irradiation **(b)** Comparison of temperature profiles of polydopamine-coated LCN and pure LCN subjected to NIR light (9.8 mW mm^{-2}). The infrared camera was used to measure the temperature change.

12. Photocontrolled deformation reversal of polydopamine-coated LCNs

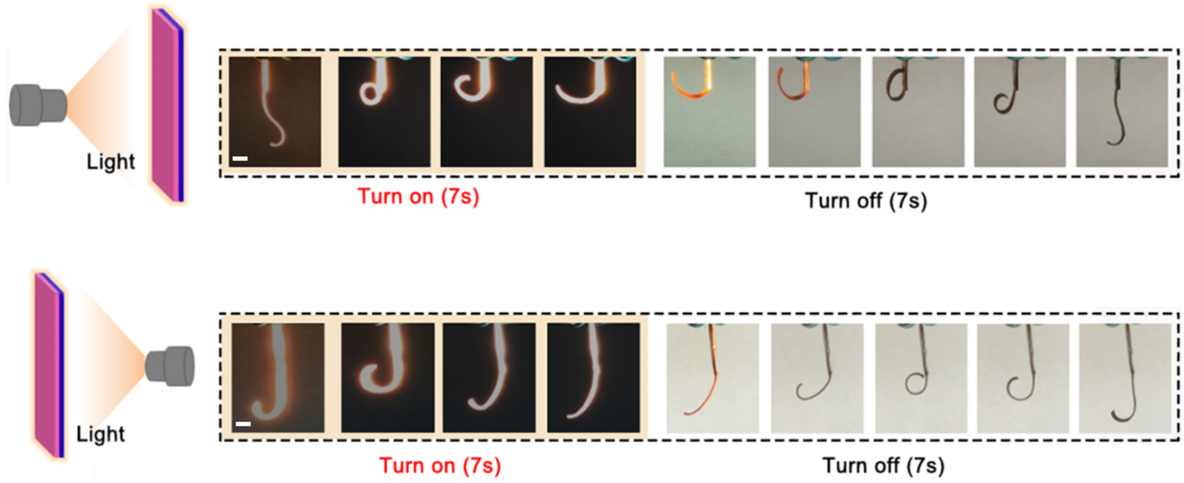

Supplementary Figure 20 |. Deformation reversal of polydopamine-coated LCN_1(20,120) actuator irradiated by NIR (12.7 mW mm^{-2}) on 20 min-crosslinked side (**top**) and 120 min-crosslinked side (**bottom**). Although the shape transformation is not exactly the same, the deformation reversal behavior in the two cases remains mostly similar, indicating that the deformation reversal behavior is mainly determined by the actuator processing, rather than the incident light direction (scale bars: 2 mm).

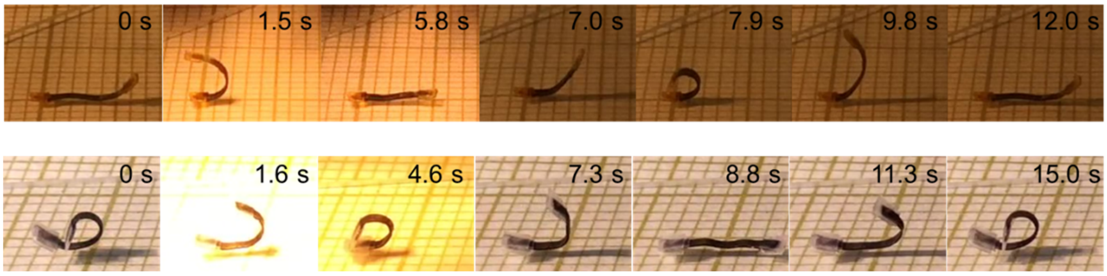

Supplementary Figure 21|. Light-induced deformation reversal behavior of polydopamine-coated LCN_1(20,120) actuator **(top)** which can bend-unbend-bend-unbend and polydopamine-coated LCN_2(1000%,400%) actuator **(bottom)** which can unbend-bend-unbend-bend during one NIR light (16.8 mW mm^{-2}) on/off cycle. Note that the graph paper in the background serves as a scale reference: ten squares per centimeter.

13. Locomotion mechanism of the wave-shaped walker

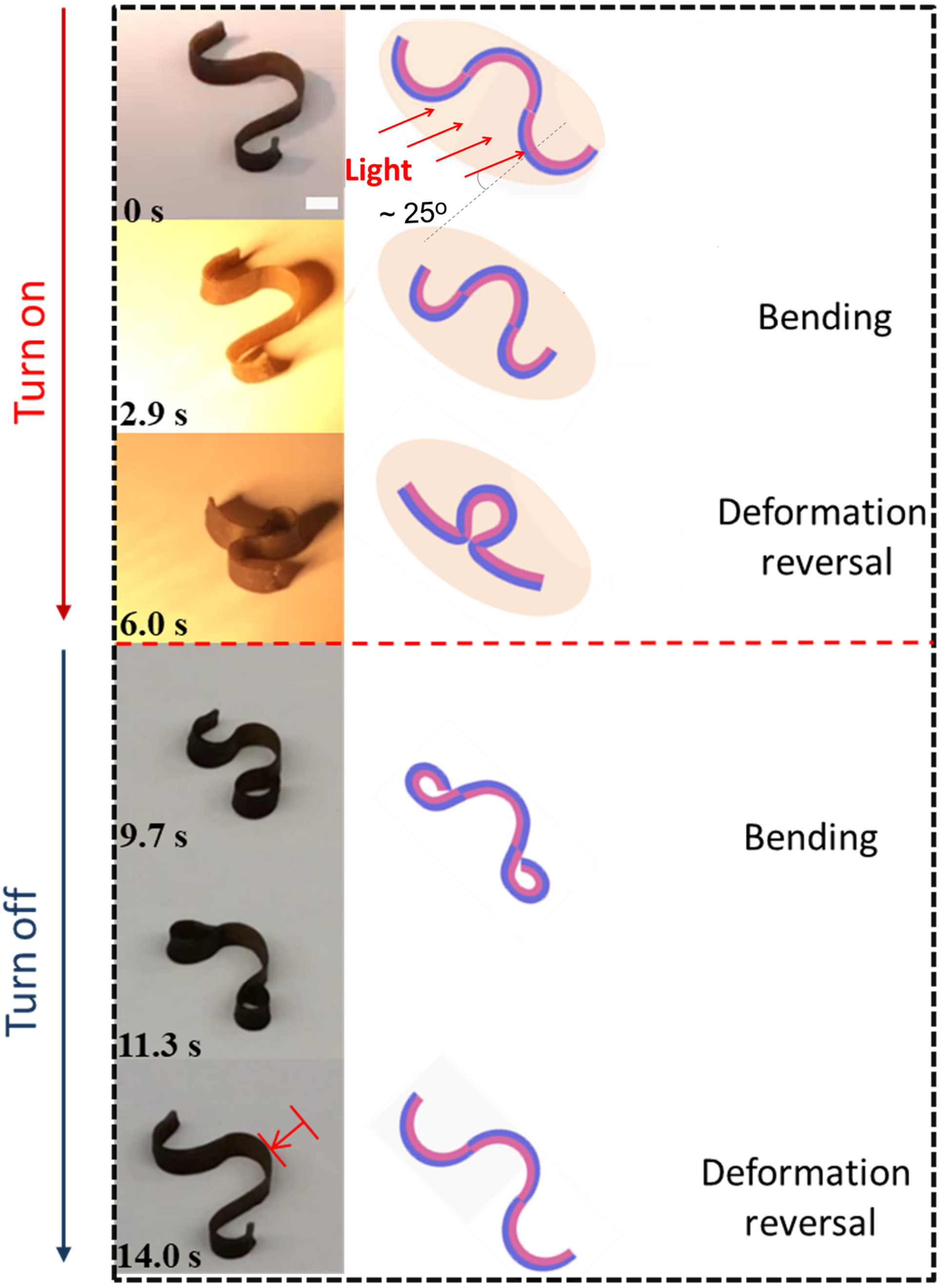

Supplementary Figure 22 |. Representative photographs and illustration showing locomotion behavior of the wave-shaped walker during one NIR light on/off cycle (scale bar: 2 mm).

14. Multi-mode motion of a single deformation-reversal LCN actuator (preparation shown in Figure 5a)

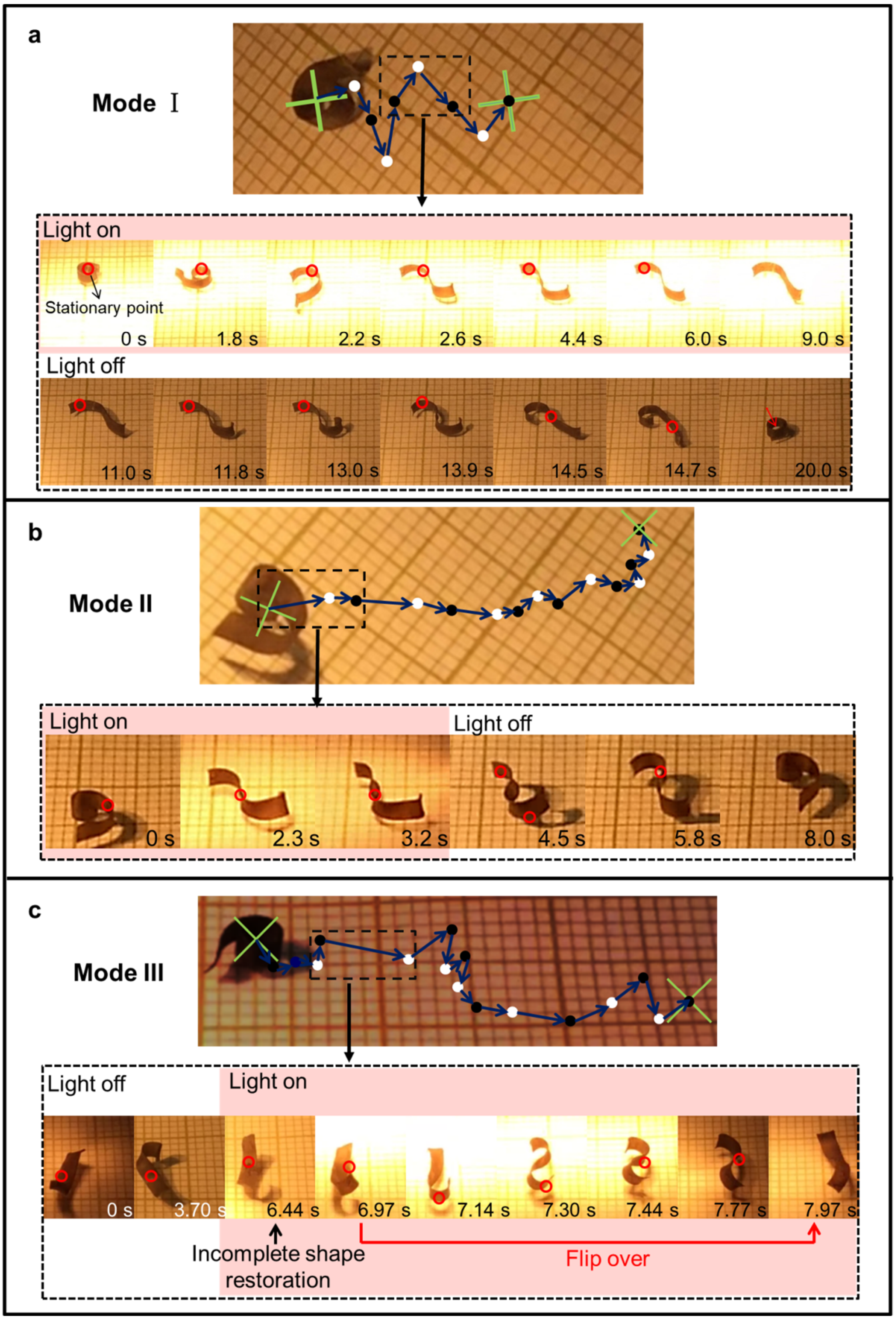

Supplementary Figure 23 |. Motion trajectory of the actuator (up) and the corresponding locomotion mode illustration through the snapshots from one light on-off cycle (down) for **(a)** Mode I (moving while rotating its body direction), **(b)** Mode II (walking based on unidirectional deformation) and **(c)** Mode III (advancing via turning over). The green cross marks the start/end point; the black and white points represent the position of the actuator when the light is turned on and off, respectively. The red circle marked in the photograph represents a stationary point, which remains fixed when the current shape evolves to the shape shown in the next photograph. Note that the graph paper in the background can be used as a scale reference: ten squares per centimeter.

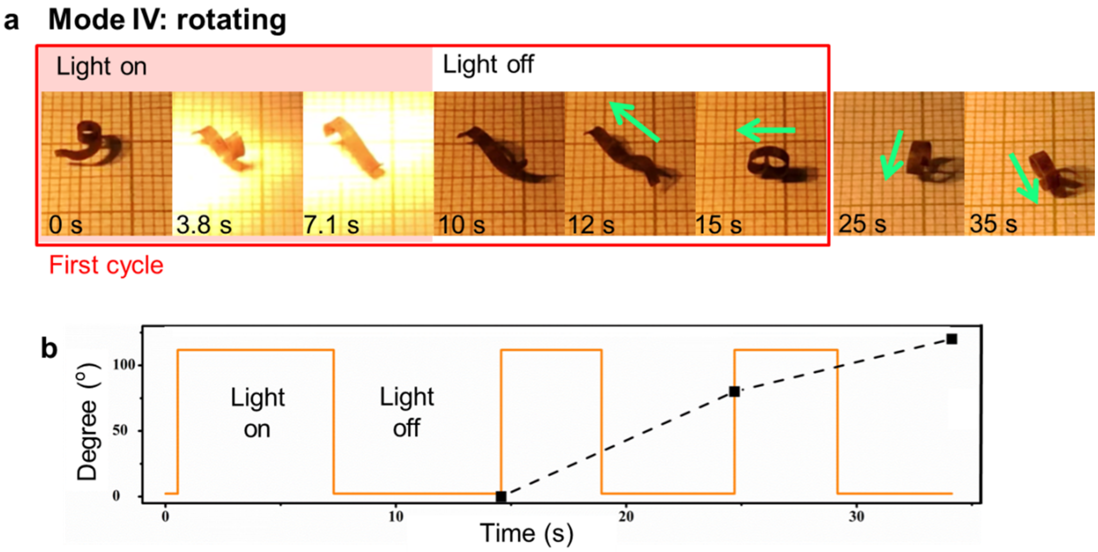

Supplementary Figure 24 |. (a) Photographs showing the first cycle of the motion Mode IV as well as the rotation behavior during light on-off cycles. **(b)** Light irradiation pattern and the corresponding rotating degrees of the actuator versus time. Light intensity used is 23.5 mW mm^{-2}. The green arrow represents the rotation direction of the actuator. Note that the graph paper in the background can be used as a scale reference: ten squares per centimeter.

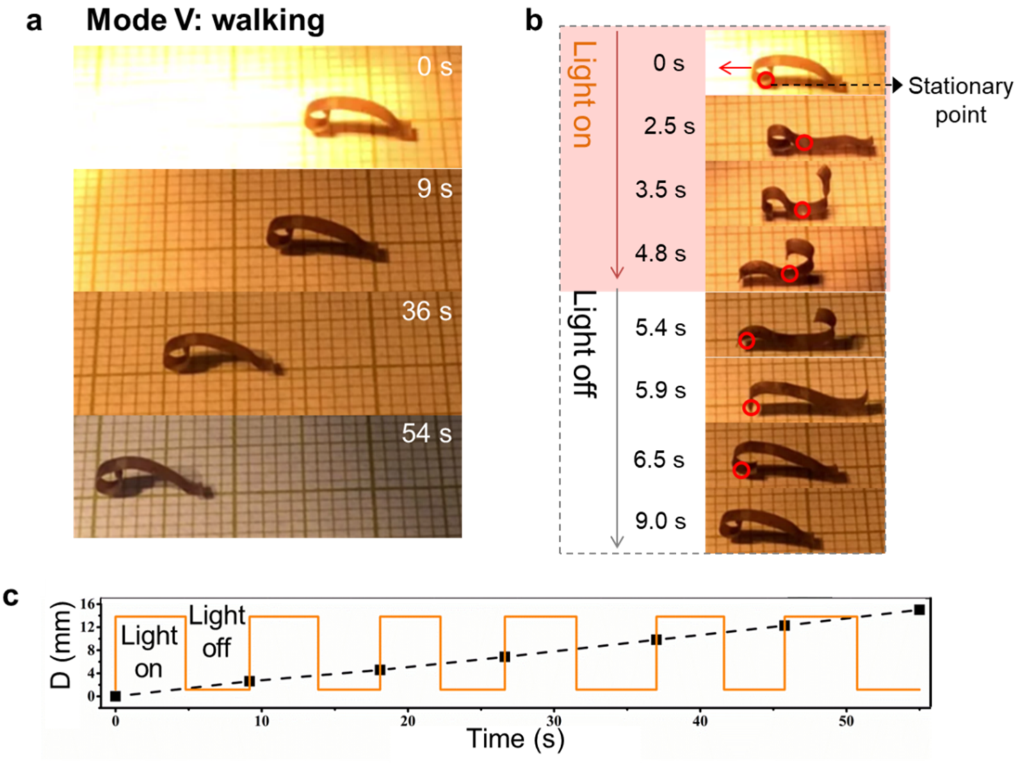

Supplementary Figure 25 |. (a) Photographs showing the motion Mode V of the actuator: walking via the unidirectional uncurling or curling motion of its left and right parts while its planar side facing parallel to the substrate. **(b)** Snapshots of the locomotion taken from the first light on/off cycle. **(c)** Light irradiation pattern and the corresponding displacement (D) versus time of the actuator. Light intensity used is 16.8 mW mm^{-2}. The locomotion speed is 16.7 mm min^{-1}. Note that the graph paper in the background can be used as a scale reference: ten squares per centimeter.

15. NMR spectra of liquid crystalline polymers (LCPs)

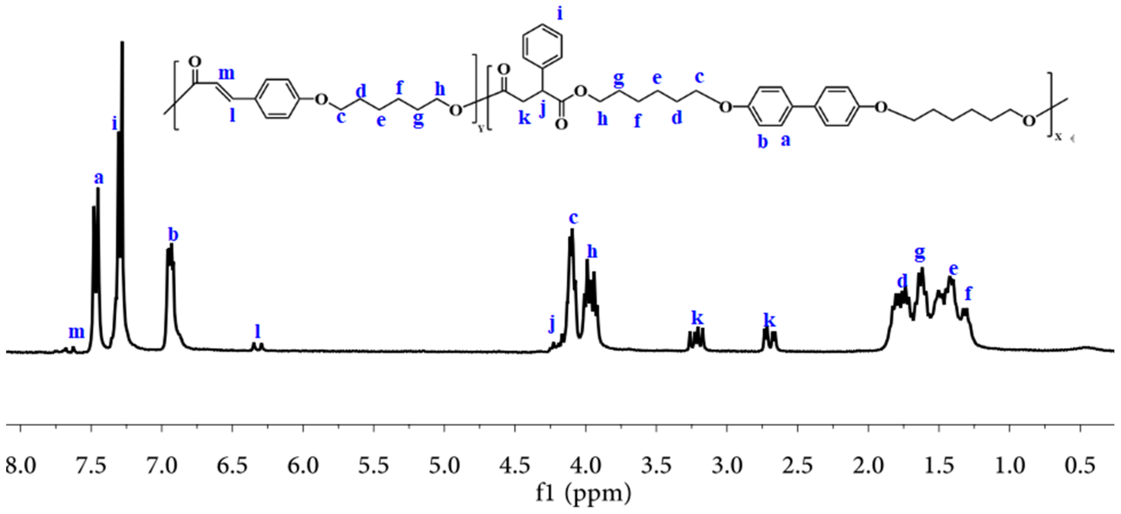

Supplementary Figure 26 |. ^{1}H NMR spectrum of LCP in $CDCl_3$

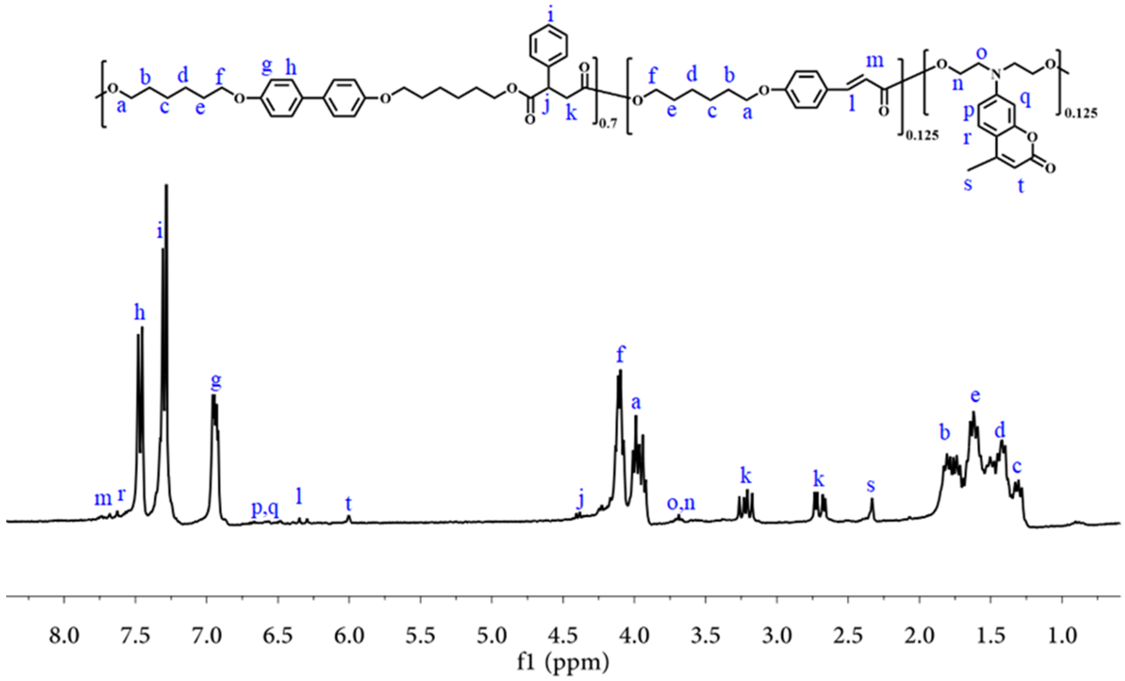

Supplementary Figure 27 |. ^{1}H NMR spectrum of the coumarin-LCP in $CDCl_3$

Supplementary Table

Supplementary Table 1. Dimensions of LCN actuators.

Figure	Sample code	Length (l, mm)	Thickness (d, mm)	Width (mm)
Supplementary Fig.2	LCN_1(20,120)	9.65	0.16	–0.65 ± 0.10
ig. 2 and Supplementary Fig.3	LCN_1(0,120)	9.63	0.17	
	LCN_1(20,120)	9.58	0.15	
	LCN_1(40,120)	9.55	0.16	
	LCN_1(60,120)	9.68	0.15	
	LCN_1(120,120)	9.52	0.16	
Supplementary Fig.4	LCN_1(0,60)	9.45	0.16	
	LCN_1(20,60)	9.48	0.15	
	LCN_1(40,60)	9.51	0.17	
	LCN_1(60,60)	9.58	0.16	
Supplementary Fig.5	LCN_1(80,60)	9.50	0.19	
	LCN_1(120,60)	9.48	0.18	
	LCN_1(120,20)	9.58	0.18	
Supplementary Fig.7	LCN_1(20,120) (0.2 mm original thick-ness)	9.48	0.09	
	LCN_1(20,120) (0.48 mm original thickness)	9.55	0.24	
	LCN_1(20,120) (54°C stretching tem-perature)	9.62	0.15	
	LCN_1(20,120) (64°C stretching tem-perature)	9.48	0.17	
	LCN_1(20,120) (800 % draw ratio)	9.35	0.11	

	LCN_1(20,120) (1000 % draw ratio)	9.43	0.10	
Supplementary Fig.10	Coumarin-LCN (120,60)	9.62	0.23	
	Coumarin-LCN (90,60)	9.62	0.24	
Fig. 3 and Supplementary Fig.14	LCN_2(0%,400%)	9.63	0.16	
	LCN2(200%,400%)	9.52	0.16	
	LCN_2(300%,400%)	9.60	0.15	
	LCN_2(400%,400%)	9.58	0.15	
	LCN_2(540%,400%)	9.67	0.14	
	LCN_2(700%,400%)	9.59	0.14	
	LCN_2(1000%,400%)	8.13	0.13	
Supplementary Fig.15	Fig.15a	14.5	0.17	2.06
	Fig.15b	16.1	0.16	1.94
Supplementary Fig.18 and 20	polydopamine-coated LCN_1(20,120)	9.49	0.15	0.62
Fig.4b and Supplementary Fig.22	Wave-shaped walker	23.06	0.18	1.36
Fig.5 and Supplementary Fig.23, 24, and 25	Multimodal actuator	13.54	0.13	1.40

Supplementary Discussion

This experiment was designed to observe the effect of only polymer chain crosslinking on the LC-isotropic phase transition of the LCN used. In order to obtain clear data on chain cross-linking density, photocrosslinking of the polymer dissolved in solution was carried out, which made it possible to monitor the increase in the photodimerization degree by ^{1}H NMR spectroscopy (Supplementary Figure 1a). The LCN samples dried from the solution, being unstretched and having various crosslinking densities, were used for the DSC measurements. The results clearly show the effect of chain crosslinking. Indeed, with increasing crosslinking density, the lower-temperature smectic-nematic phase transition peak in the uncrosslinked polymer is gradually weakened and suppressed to finally emerge with the nematic-isotropic transition peak. This leads to a broad nematic-isotropic phase transition and a broad thermo-mechanical or photomechanical actuation region (smectic-nematic transition does not provide a significant actuation). In the same time, the overall order-disorder phase transition enthalpy decreases with increasing the crosslinking. These results imply that higher chain crosslinking density impose greater chain topological constraints that have an effect to hinder the formation of smectic layer. This effect is similar to what was revealed in a previous study[S2]: the mechanical stretching of the LCN can suppress the smectic ordering and give rise to a broad actuation temperature region.

The effect of chain crosslinking and chain stretching on LC behavior is a complex issue and depends on many factors (the LCN used, the order state of the sample during crosslinking, the crosslinking chemistry, polymer compositions and crosslinking agents of different chem-

ical structures, to name a few).[S3-S7] Nevertheless, in the present study, experimental evidence shows that both chain stretching and chain crosslinking can affect the order-disorder phase transition region. By applying different crosslinking densities and/or different chain stretching to the two sides of an LCN strip, the actuation temperature range as well as the resulting thermomechanical or photomechanical forces on the two sides can be “designed” to differ from each other. This is the basis of the desynchronized actuator with deformation reversal capability.

Supplementary Methods

Materials

4,4'-Dihydroxybiphenyl (Aldrich, 97%), p-coumaric acid (Aldrich, 99%), 6-chlorohexanol (Aldrich, 96%), phenylsuccinic acid (PSA) (Aldrich, 98%), tris(hydroxymethyl) amino-methane (Aldrich, 99%), dopamine hydrochloride (Aldrich), 3-aminophenol (Aldrich, 99%), ethyl acetoacetate (Aldrich, 98%) and 2-cholorothanol (Aldrich, 99%) were used directly without further purification.

Synthesis of liquid crystalline polymers (LCPs)

The monomers, 4,4'-bis(6-hydroxyhexyloxy)biphenyl (BHHBP) and 4-(6-Hydroxy-hexyl -oxy)cinnamic acid (6HCA), were synthesized using the previously reported method.[S8,S9] BHHBP (1159.6 mg, 3 mmol), PSA (582.5 mg, 3 mmol) and 6HCA (211.4 mg, 1 mmol) were put into a 100-mL three-necked flask, which was equipped with nitrogen inlet and nitrogen outlet, a mechanical stirrer, as well as a distillation trap connected to a vacuum line. Then, the Zn $(Ac)_2$ (3.9 mg , 0.2 wt%) and Sb_2O_3 (5.9 mg, 0.3 wt%) acted as catalysts were added into the flask. In order to remove air, the three-neck flask was evacuated and filled with N_2 several times, and then the reaction was carried out at 150° C under N_2 flow with mechanically stirring. After 4 h, the system was further heated to 180 °C with a high vacuum of 30-50 Pa and kept for another 4 h. Finally, the LCP sample was collected after cooling of the flask to room temperature. The product was purified by precipitating/dissolving in methanol/chloroform 3 times and dried under vacuum below 45 °C to obtain LCP as white solid. ^1H NMR is shown in **Supplementary Figure 26**.

Synthesis of coumarin-LCP and coumarin-LCN actuator with nematic phase

7-(Bis(2-hydroxyethyl)amino)-4-methylcoumarin (BHAMC) were synthesized using the previously reported method.[S10,S11] BHHBP (1159.6 mg, 3 mmol), PSA (679.6 mg, 3.5 mmol), BHAMC (131.6 mg, 0.5 mmol) and 6HCA (105.7 mg, 0.5 mmol) were put into a 100-mL three-necked flask and polymerized through the similar steps as mentioned above. The $Zn(Ac)_2$ (4.1 mg , 0.2 wt%) and Sb_2O_3 (6.2 mg, 0.3 wt%) were added into the flask to act as catalysts. After polymerization, the coumarin-LCP was purified by precipitating/dissolving in methanol/chloroform for 3 times and dried under vacuum at 45 °C. ^{1}H NMR and the XRD results are shown in **Supplementary Figure 27** and **Supplementary Figure 9**, respectively.

The compression-molded polydomain coumarin-LCP film (0.48 mm in thickness) was stretched uniaxially at 50 ºC in water to λ=400%. The stretched film with the monodomain of uniaxial orientation of mesogens was asymmetrically photocrosslinked to obtain coumarin-LCN(120,60) and coumarin-LCN(90,60) (**Supplementary Figure 10**).

Characterizations

^{1}H NMR spectra of the synthesized LCPs were recorded at room temperature on a Bruker Avance-III 300 NMR apparatus. The UV-vis absorption spectra were recorded on a spectrophotometer (Cary series UV-Vis-NIR, Agilent Technologies) in the wavelength range of 200-1000 nm. Fourier transform infrared (FI-IR) spectra of LCN and polydopamine-coated LCN were recorded on a Nicolet-IS10 (Thermo Electron Co., USA) spectrometer. The mechanical property and Young's modulus of LCN actuators were measured using an Instron5965 universal testing system at a strain rate of 5 mm min^{-1}. The morphology of polydopamine-coated LCN actuator was examined using a Hitachi S-4700 field-emission-gun

scanning electron microscope (SEM) operating at 1.0 kV to10.0 kV. For photocrosslinking, an OmniCure@Series 1000 UV lamp with a 320-480 nm filter (320 nm, 180 mW.cm^{-2}, the UV light intensity was measured using a photometer set at 320 nm) was utilized to irradiate the LCPs. For light-fueled deformation and locomotion, the Flash Torch, Wicked Lasers (provides >90% of the light in the NIR region), was used to irradiate the polydopamine-coated LCN strips. The light intensity was obtained using a laser power meter (TUNER model, gentec-co company, Canada). NIR-induced temperature change of the sample was monitored using an infrared camera (Testo 875i). Thermal phase transitions of the samples were measured using a TA Q200 differential scanning calorimeter (DSC) in the temperature range of -20 ºC to 120 ºC at a rate of 10 ºC min^{-1}. The measurements were taken with about 5 mg sample under a nitrogen atmosphere (flow rate of 50 mL min^{-1}) and the data were recorded on the first cooling and second heating scan. 2D-XRD patterns were collected on a Bruker AXS Nanostar system equipped with a Microfocus Copper Anode at 45 kV/0.65 mA, Montel optics and a VANTEC 2000 2D detector at 11.15 cm distance from the samples calibrated with a Silver Behenate standard. The exposure time was 200 seconds.

A dynamic mechanical analyser (DMA Q800, TA Instruments) was used to determine the actuation strain generated by LCNs. The tests were performed in controlled force mode with a fixed force of 0.01 N. The samples were first cooled from 90 °C to 20 °C at 3 °C min^{-1} and kept isothermal for 2.00 min, and then re-heated to 90 °C at a rate of 3 °C min^{-1}. The strain is defined as the $(L-L_{iso})/L_{iso}$, where L is the extended length of the sample after cooling to LC phase, L_{iso} is the contracted length in isotropic phase. In addition, to investigate and compare the deformation of different samples, we focused on the shape change of LCNs when cooling

in water from 75 ºC to 40 ºC within ~8 min (~4.4 ºC min^{-1}) in all the thermal-responsive actuation.

Supplementary References

S1. Li, Z. *et al.* Polydopamine coated shape memory polymer: enabling light triggered shape recovery, light controlled shape reprogramming and surface functionalization. *Chem. Sci.* **7**, 4741-4747 (2016).

S2. Yang, R & Zhao, Y. Multitemperature memory actuation of a liquid crystal polymer network over a broad nematic–isotropic phase transition induced by large Strain. *ACS Macro Lett.* **7**, 353-357 (2018)

S3. Küpfer, J., Nishikawa, E. & Finkelmann, H. Densely crosslinked liquid single-crystal elastomers. *Polym. Adv. Technol.* **5**, 110-115 (1994).

S4. Wang, Y. & Burke, K. A. Phase behavior of main-chain liquid crystalline polymer networks synthesized by alkyne–azide cycloaddition chemistry. *Soft Matter,* **14**, 9885-9900 (2018).

S5. Burke, K. A., Rousseau, I. A. & Mather, P. T. Reversible actuation in main-chain liquid crystalline elastomers withvarying crosslink densities. *Polymer,* **55**, 5897-5907 (2014).

S6. Zubarev, E. R., Talroze, R. V., Yuranova, T. I., Vasilets, V. N. & Platé, N. A. Influence of crosslinking conditions on the phase behavior of a polyacrylate-based liquid-crystalline elastomer. *Macromol. Rapid Commun.* **17**, 43-49 (1996).

S7. Saed, M. O. *et al.* Thiol-Acrylate Main-Chain Liquid-Crystalline Elastomers with TunableThermomechanical Properties and Actuation Strain. *J. Polym. Sci. B Polym. Phys.* **55**, 157-168 (2017).

S8. Yang, R. & Zhao, Y. Non-uniform optical inscription of actuation domains in a liquid crystal polymer of uniaxial orientation: an approach to complex and programmable shape changes. *Angew. Chem. Int. Ed.* **56**, 14202-14206 (2017).

S9. Yang, R., Chen, Li., Ruan, Chao., Zhong, H. Y. & Wang, Y. Z. Chain folding in main-chain liquid crystalline polyesters: from π–π stacking toward shape memory. *J. Mater. Chem. C,* **2**, 6155-6164 (2014).

S10. Huang, Q., Bao, C., Ji, W., Wang, Q.& Zhu, L. Photocleavable coumarin crosslinkers based polystyrene microgels: phototriggered swelling and release *J. Mater. Chem.,* **22**, 18275-8282 (2012).

S11. Peyret, A. et al Polymersome Popping by Light-Induced Osmotic Shock under Temporal, Spatial, and Spectral Control. *Angew. Chem. Int. Ed.*, **56**, 1566-1570 (2017).
