# Supplementary material for: Desynchronized liquid crystalline network actuators with deformation reversal capability
Source: Nat Commun. 2021 Jan 27;12:624. doi: 10.1038/s41467-021-20938-6 (PMC7840968; doi:10.1038/s41467-021-20938-6)
Supplement: Supplementary file 3 — Description of Additional Supplementary Files [file 41467_2021_20938_MOESM3_ESM.pdf]

Description of Additional Supplementary Files

File Name: Supplementary Movie 1
Description: Deformation reversal of aligned LCN_1(20,120), LCN_1(40,120), LCN_1(60,120) actuators during cooling processes, and reversible deformation reversal recorded in one cooling/heating cycle for LCN_1(20,120).

File Name: Supplementary Movie 2
Description: Deformation reversal of aligned LCN_2(540%,400%), LCN_2(700%,400%) and LCN_2(1000%,400%) actuators during cooling.

File Name: Supplementary Movie 3
Description: Twisting-untwisting bidirectional deformation of a -45° aligned LCN_1(60,120) actuator during cooling.

File Name: Supplementary Movie 4
Description: Unwinding-winding bidirectional deformation of a +45° aligned LCN actuator during cooling.

File Name: Supplementary Movie 5
Description: Light-induced deformation reversal behavior of polydopamine(PDA)-coated LCN actuators: PDA-coated LCN_1(20,120) and LCN_2(1000%,400%) actuators.

File Name: Supplementary Movie 6
Description: Light-driven micro-walker of polydopamine-coated LCN_1(20,120).

File Name: Supplementary Movie 7
Description: Light-driven shape change and locomotion of a wave-shaped deformation-reversal actuator.

File Name: Supplementary Movie 8
Description: Light-driven multimodal locomotion of a single deformation-reversal actuator upon different light irradiation patterns: locomotion mode I, II and III.

File Name: Supplementary Movie 9
Description: Light-driven multimodal locomotion of a single deformation-reversal actuator upon different light irradiation patterns: locomotion mode IV and V.
